# The development of translational tasks for preclinical psychedelic research in psychiatry

**DOI:** 10.1177/02698811251365167

**Published:** 2025-08-26

**Authors:** Emma SJ Robinson

**Affiliations:** School of Physiology, Pharmacology and Neuroscience, University Walk, Bristol, UK

**Keywords:** Psychedelics, preclinical psychiatry, animal models, major depressive disorder

## Abstract

Psychiatric disorders represent a particularly challenging area of medicine to study using non-human animals. They arise from a complex interaction between genetic and environmental factors and are diagnosed using clinical interviews and diagnostic criteria based largely on self-reported symptoms and with significant heterogeneity seen within patient populations. Anxiety and major depressive disorder are the most diagnosed psychiatric disorders, with prescriptions of antidepressants exceeding 80 million in the UK in 2024. Despite more than 70 years of research, we do not understand how antidepressants achieve their effects on mood. With a resurgence in interest in developing new treatments based on clinical advances in the psychedelic field, there is a pressing need for translational methods to study the underlying mechanisms and provide reliable predictions of clinical effects. Human studies are limited in terms of the resolution of current imaging methods and the depth of mechanistic interrogation which can be achieved. Studies involving non-human animals offer an important avenue to understand how psychedelics alter behaviour and the underlying mechanisms mediating these effects, as well as playing a critical role in the development of new drugs. However, the value of these studies in terms of delivering outcomes for patients will only be achieved if the methods used have clinical relevance. In this perspective article, I consider whether ‘behavioural biomarkers’, and their translation to animal tasks, could achieve this much-need approach.

## Introduction and context

Achieving translational validity, defined here as how behavioural readouts in animals translate to human psychiatric symptoms, was central to Roland’s philosophy when conducting experiments in animals, which included his seminal self-administration studies on drugs of abuse in baboons (Brady and Griffiths, 1976). Depending on the field, how we define translational validity can differ. For much of medicine, blood, physiological or imaging biomarkers can provide a trans-species method to quantify changes which reflect disease development and treatment effects. In psychiatry, this is more challenging as the symptom domains used to define the disorders use clinical interviews and diagnostic criteria based largely on self-reported measures. The nature of these symptoms is also very much defined by uniquely human experiences and the ability to articulate these verbally. It is impossible to directly relate these subjective experiences to behaviours in non-human animals, and so traditional methods used to study psychiatric disorders tend to be influenced by anthropomorphic interpretations. For example, the forced swim test (FST) and tail suspension test (TST; Porsolt et al., 1977, 1978; Steru et al., 1985), developed to screen antidepressants acting via mechanisms which increase synaptic monoamines, are described as quantifying behavioural despair and are aligned with the human experience of ‘hopelessness’. The other commonly used readout for major depressive disorder (MDD) research is the sucrose preference test (SPT; Willner et al., 1987), where an animal’s ability to distinguish a low-concentration sugar solution from water is aligned with the human experience of a loss of interest in rewarding activities. Although these tasks are sensitive to chronic stress manipulations and respond to antidepressants, little is known about how the mechanisms mediating these behaviours in animals relate to the mechanisms which cause MDD symptoms and/or are targeted by antidepressants. When considering translational validity, these approaches have significant limitations. The FST/TST is sensitive to chronic stress manipulations but data for other risk factors for MDD are more mixed (Commons et al., 2017; De La Garza et al., 2005; Stupart et al., 2023). Drug treatments which lead to changes in the main readout, immobility time, seem to only achieve predictive validity if their mechanism of action involves increasing synaptic concentrations of monoamines. Drugs acting through other mechanisms may change immobility time, but the predictive value of these findings for patients is less reliable (Molendijk and de Kloet, 2015). In relation to the SPT, when a similar test was performed in people, no deficits were observed in MDD despite subjective symptoms of anhedonia (Dichter et al., 2010). This may relate to differences in the domains of reward processing being tested using these different methods (Thomsen, 2015). Questionnaire-based measures suggest that reward-related impairments in MDD are not related to the ability to experience reward but are more about the anticipation of reward based on past experiences, that is, involving learning and memory (Slaney et al., 2018). Relevant to the recent interest in psychedelic drugs as novel therapeutics are also animal models used to quantify hallucinations and the psychedelic effects of drugs (Alexander et al., 2024). The most often reported method to study the hallucinatory effects of a drug is the head twitch response (HTR, mice) and wet dog shakes (WDSs, rat), but what underlies these behaviours is poorly understood (Halberstadt and Geyer, 2018).

With increasing pressure to replace studies in living animals with non-animal methods and with limited translation of fundamental neuroscience to clinical benefits, animal models have received much of the criticism for these failures. Rather than interpreting these failures as a general lack of relevance of animal models for preclinical psychiatry, this article argues for greater investment in the development and validation of new behavioural approaches. Methods to study the function of the brain have seen huge advances in recent decades. Providing ever more detailed insights into micro and macro neural circuits, genetic and molecular mechanisms, these approaches offer huge potential but are currently limited by the lack of advances in behavioural approaches. Almost all current preclinical studies related to psychiatric disorders still rely on what are ultimately pharmacological screening tools designed to be sensitive to drugs which have efficacy in patients, for example, FST or SPT for antidepressants or psychostimulant-induced hyperlocomotion to study antipsychotics. The use of these approaches for mechanistic studies and the over-interpretation of the findings in terms of their relevance to psychiatric disorders risks continuing to undermine the field, particularly when fuelled by the current hype associated with psychedelics.

## Why studies using traditional behavioural tasks may have limited translational validity for MDD

Studies in rodents have been the foundation of research into the mechanisms which underlie the effects of antidepressant drugs and the identification of novel drug targets. Unfortunately, despite huge financial investment, the outcome for patients remains largely unchanged. Even with the introduction of the rapid-acting antidepressant (RAADs) and N-methyl-D-aspartate (NMDA) antagonist, ketamine, there are still many patients for whom new treatments are needed. So why have so few of the findings from rodent models translated into clinical benefits? For the last decade or more, concerns have been raised about the translational relevance of the behavioural readouts used in animal studies. Behavioural tests are currently the only outcome measure which can be related to clinical symptoms, but what may be seen as depression-like behaviours in animals could involve different underlying mechanisms. The justification used for the relevance of these tasks comes mainly from studies involving chronic stress manipulations and the generation of a behavioural phenotype which is reversed with conventional antidepressant treatment. To a certain degree, this would seem justified as stress is an important risk factor for MDD, and sensitivity to conventional antidepressant treatments would suggest modulation of a relevant causal mechanism. However, it may also be that the stress-induced deficits generated using short-term moderate to severe stressors in animals involve different underlying mechanisms than those involved in human MDD.

The development of MDD involves precipitating factors, but these are more diverse than stress and can include early life adversity, chronic inflammation and co-morbid disorders, including chronic pain. While chronic stress in rodents generates a phenotype in the FST/TST and SPT, this is not reliably detected with other models, including maternal separation (Stupart et al., 2023) or chronic interferon alpha treatment (De La Garza et al., 2005). This not only matters for studies investigating aetiological mechanisms, but also for studies investigating the mechanisms of action of current antidepressants and identifying novel drug targets. If the behavioural readouts involve different underlying mechanisms, then the findings may only be relevant to these animal models and not engage mechanisms relevant to patients. In addition to the findings with conventional antidepressants, the RAAD ketamine reduces immobility in the FST/TST and increases sucrose preference, but the picture with psilocybin is less clear (Alexander et al., 2024), and non-efficacious NMDA antagonists also alter immobility time in the FST (Viktorov et al., 2022). The predictive validity of these tasks may therefore be limited to certain pharmacological mechanisms. Since we have developed tasks which we suggest have greater translational validity, we have also noted differences between antidepressant doses and efficacy in these different behavioural tests (Anderson et al., 2024). Doses of antidepressants which are effective in our translational tasks are significantly lower than those usually reported with traditional models and align more closely with clinical doses and associated receptor occupancy. Identifying dose equivalence between species is difficult, as the pharmacokinetic data needed to achieve this are often not available. There is a tendency to use higher doses in animals due to their higher metabolic rate and impacts on metabolism and excretion. However, doses used in traditional behavioural tests for depression-related behaviours are often more than 10- or even 100-fold greater (Anderson et al., 2024). These doses will achieve higher levels of receptor occupancy than occur in patients and engage off-target receptors, leading to downstream effects which may not be directly relevant to MDD. Unfortunately, concerns about the translational validity of these traditional animal models, and the hypotheses generated from them in terms of novel mechanisms, have recently been borne out with the failure of pro-neurotrophic drugs to demonstrate efficacy in patients despite compelling preclinical evidence (Alto Neuroscience, 2024).

## Development of translational behavioural tasks for rodents based on ‘behavioural biomarkers’ in MDD patients

Not all clinical and experimental medicine research associated with MDD uses self-reported measures, with a growing body of literature based on the use of objective behavioural readouts, most of which are delivered as computer-based tasks. These can be integrated with imaging methods providing insights into underlying neural circuits (Hamani et al., 2011; Ressler and Mayberg, 2007, Figure 1). In the absence of suitable imaging or blood biomarkers, behavioural readouts are currently the only viable approach for developing novel preclinical methods with improved translational relevance for investigating fundamental biology or evaluating the potential efficacy of novel treatments, including psychedelics.

**Figure 1.**
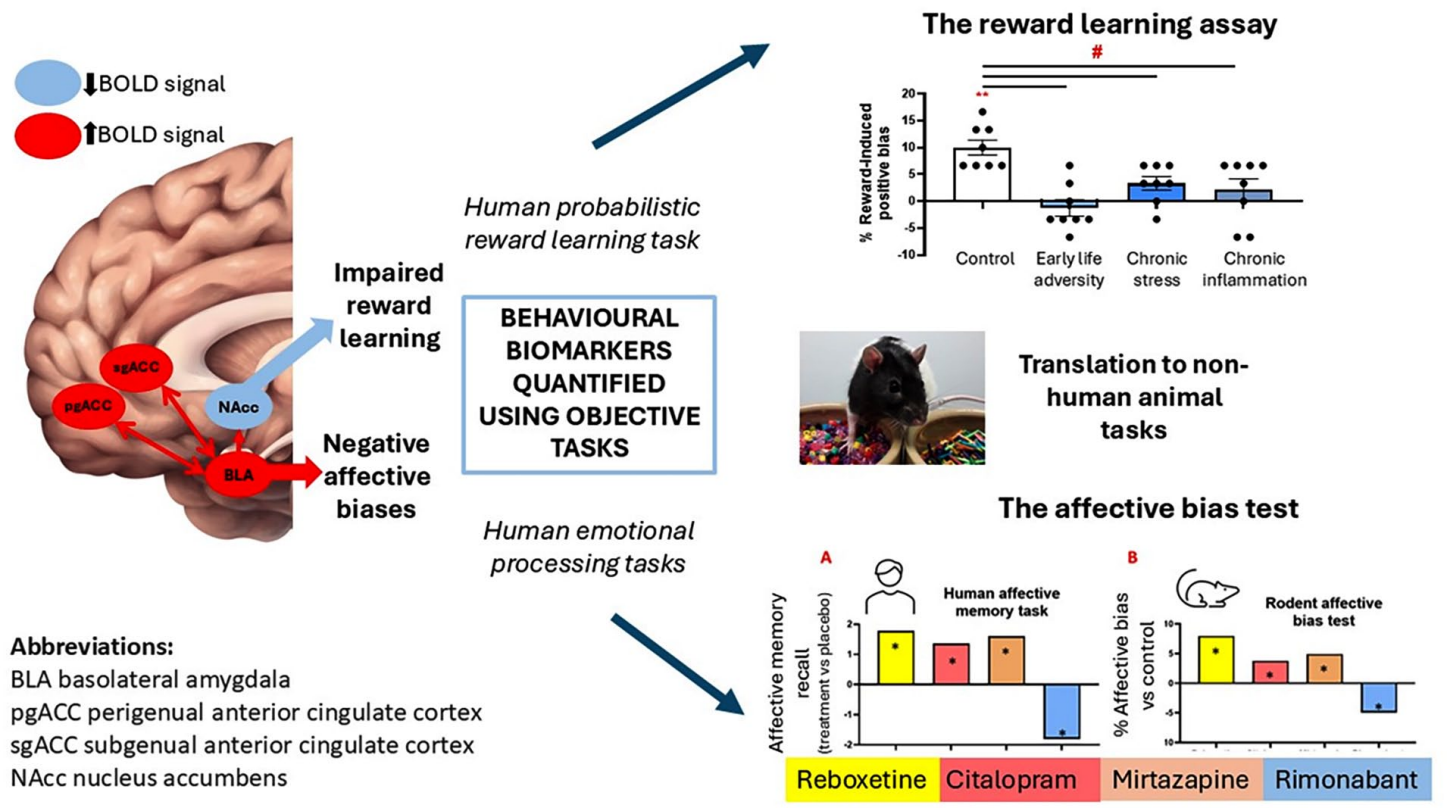
Translational tasks developed to quantify impairments in reward learning and affective bias modification in rodents. MDD is associated with negative affective bias influencing emotional processing and reward learning. These changes can be quantified objectively using emotional memory tasks or a probabilistic reward learning task, respectively. Integrating these tasks with fMRI has identified activity changes in key brain regions in MDD patients, which are remediated with effective antidepressant treatment (Pizzagalli and Roberts, 2022). The rat reward learning assay (RLA) can detect reward learning impairments in animal models of depression associated with different risk factors for MDD and acute pharmacological manipulations of affective state quantified using the affective bias test (ABT) show similar findings with antidepressant (reboxetine (noradrenaline re-uptake inhibitor), citalopram (serotonin re-uptake inhibitor), mirtazapine (noradrenaline and serotonin receptor antagonist)) and pro-depressant (rimonabant (cannabinoid_1_ receptor inverse agonist)) drugs to those observed in a human affective memory task. Both tasks are based on associative learning and memory and the pairing of specific cues (digging substrates) with rewards (Hinchcliffe et al., 2024a). Biases in the reward-associated memory are modified by affective state at the time of learning and can also be attenuated at retrieval by RAADs (Stuart et al., 2013, 2015; Hinchliffe et al., 2024b). **p<0.01 one-sample t-test vs 0% bias, #p<0.05 pairwise comparison control vs depression model.

Where studies in MDD patients use computer-based tasks to provide an objective assessment of cognitive and emotional behaviours, impairments in key behavioural domains have been identified. Patients exhibit changes in what is sometimes referred to as ‘hot’ and ‘cold’ cognition (for further discussion, see Elliott et al., 2011; Roiser et al., 2012; Roiser and Sahakian, 2013). Arising from these studies is the observation that patients with MDD exhibit negative affective biases, a change in ‘hot’ cognition. Affective biases arise when emotional states influence cognition, and negative affective biases impact learning and memory, decision-making and attention in MDD patients (Elliott et al., 2011; Gotlib and Joormann, 2010; Leppänen, 2006; Roiser et al., 2012; Roiser and Sahakian, 2013). For studies in animals, these objective methods and ‘behavioural biomarkers’ provide an opportunity to use reverse translation and develop and validate novel behavioural methods for rodents. While the tasks used in patients are mostly based on language or facial recognition, the underlying construct of affective biases can be integrated with cue-based behavioural tasks for rodents. The aim with these tasks is to achieve parity in terms of underlying neuropsychological mechanisms but also recognise species differences, tailoring the task as much as possible to the animal’s natural behaviours. This helps to reduce potential confounds of over-training and other factors, such as the need to use food or water restriction to generate high motivational states. Figure 1 illustrates two areas of impairment observed using objective tasks in MDD patients and how these have been translated into methods for rodents and with examples of data illustrating their translational validity.

Impacting on cognitive domains of attention, decision-making and learning and memory, negative affective biases are thought to contribute to the development and perpetuation of MDD. Using acute and short-term treatments with antidepressants, for example, reboxetine (noradrenaline re-uptake inhibitor), citalopram (serotonin re-uptake inhibitor), mirtazapine (noradrenaline and serotonin receptor antagonist), and quantifying changes in emotional processing, studies in healthy volunteers suggest these drugs can induce a positive shift facilitating more positively biased cognition (see Pringle et al., 2011 for full details of drugs tested in different emotional processing tasks and findings from patients and healthy volunteers). These effects are seen in the absence of any self-reported changes in mood and underpin the neuropsychological hypothesis of antidepressant efficacy, which posits that treatment effects involve modulation of the neurochemical environment to facilitate more positive emotional processing (Harmer et al., 2017). This interaction between biochemical and experience-dependent factors could explain the delayed onset of action of conventional antidepressants with efficacy dependent on new experiences, which are now positively biased, and build over time to reverse the impacts of the disease-related negative affective biases (Harmer et al., 2017). RAADs are hypothesised to act through different mechanisms modulating affective biases associated with past experiences, leading to a more rapid reversal of the influence of negative affective biases on mood (Godlewska and Harmer, 2021; Stuart et al., 2015). Using fMRI, key brain regions linked to emotional processing and reward have been shown to have altered activity in MDD (Pizzagalli and Roberts, 2022, Figure 1). This has led to a focus on hyperactivity in circuits involving the subgenual cingulate cortex and amygdala and hypoactivity in regions associated with reward processing, such as the nucleus accumbens (Hamani et al., 2011; Pizzagalli and Roberts, 2022).

Loss of interest in rewarding activities is a core symptom of MDD, and studies in patients have found impairments using objective measures of reward learning. In a probabilistic reward learning task, participants are required to respond to brief cues and healthy subjects develop a reward-induced bias, that is, they bias their responding to cues associated with higher probabilities of reward (Pizzagalli et al., 2005). This aspect of reward-related cognition seems to be most closely aligned with anhedonia and the loss of interest in rewarding activities and may be more relevant to MDD than measures of reward sensitivity.

## Rodent models of affective biases and impairments in reward learning

Building from the underlying neuropsychological concepts of human emotional processing tasks and methods to quantify reward learning, behavioural tasks for rodents have been developed. The first demonstration of an affective bias in non-human animals, published in 2004 by Harding et al. (2004), used a judgement bias task (JBT). This measure of affective state-induced biases in decision-making requires animals to be first trained to discriminate between two distinct auditory cues predicting positive or negative/less positive outcomes. Using ambiguous, intermediate cues to probe affective-state induced decision-making, animals in a putative negative affective state were found to make more pessimistic choices (Hales et al., 2014). Further characterisation of this model has found consistent phenotypic changes in ambiguous cue interpretation consistent with the prediction that this method can quantify affective biases relevant to negative emotional states (Hales et al., 2014). Pharmacological studies find more mixed results, although largely consistent with their predicted effects on mood (Neville et al., 2020). The time course of effects differs from human ambiguous cue interpretation tasks with acute conventional antidepressant treatments inducing more positive emotional processing in healthy volunteers and patients, but the same treatments require chronic administration in rats (Hales et al., 2014). RAADs were found to induce more optimistic choices following acute administration, and these effects were localised to the medial prefrontal cortex (mPFC), suggesting they may involve similar underlying neural circuits as affective bias modification in humans (Hales et al., 2020). Although the JBT exhibits some improved translational validity versus traditional methods, extended training periods and high numbers of trials may lead to confounds associated with procedural learning.

The affective bias test (ABT) is based on a more naturalistic bowl-digging task where animals are trained to associate specific digging substrates with food rewards (Hinchcliffe et al., 2024a). These methods require shorter periods of training, fewer trials and animals can learn individual substrate-reward associations within a single session. To quantify affective state (or drug treatment) induced biases, animals undergo independent substrate-reward association pairing sessions on different days and under different treatment conditions, for example, affective state manipulation versus control condition.^1^ To quantify the arising affective bias, a choice test is used where the previously rewarded substrates from each of the pairing sessions are presented at the same time and the animals’ choices are recorded. Using different pharmacological and psychosocial manipulations of affective state, the ABT is sensitive to both antidepressant and pro-depressant manipulations with a high degree of similarity to studies in healthy volunteers using the same acute treatments (Figure 1, Stuart et al., 2013, 2017). Comparing the effects of acute doses of conventional and RAADs in the ABT, conventional antidepressants were found to positively bias new experiences consistent with the neuropsychological hypothesis of antidepressant efficacy (Stuart et al., 2015). By contrast, RAADs were found to modulate affective biases associated with past experiences, an effect that might explain their more rapid onset of action (Stuart et al., 2015). Interestingly, when the serotonergic psychedelic, psilocybin, was tested using the different ABT protocols, it was shown to both positively bias new experiences and attenuate negative biases associated with past experiences (Hinchcliffe et al., 2024b). The sustained effects of RAADs are a particularly intriguing finding as this suggests effects persist beyond any direct pharmacological action and involve longer-term plasticity changes. Using the ABT, and testing animals 24 h post-treatment with ketamine or psilocybin, the negative affective biases associated with past experiences were found to now be retrieved with a relatively more positive affective valence (Hinchcliffe et al., 2024b). Using a cue reactivation protocol suggested these effects were cue-dependent, consistent with a re-learning effect and driven by experience-dependent plasticity (Hinchcliffe et al., 2024b). This was further supported by evidence that the 24 h effect was blocked by a protein synthesis inhibitor (Hinchcliffe et al., 2024b). Using targeted brain manipulations, modulation of affective biases has been shown to involve both the mPFC and amygdala, with the effects of RAADs localised to the mPFC (Stuart et al., 2015). Excitotoxic lesions to the amygdala prevented the antidepressant venlafaxine from generating a positive affective bias, while infusions of ketamine into mPFC replicated the systemic effects in terms of attenuating a negative bias associated with past experiences (Stuart et al., 2015). Studies in the early life adversity model of depression also found evidence of vulnerability to corticosterone-induced negative affective biases (Stuart et al., 2019). Taken together, these findings suggest the ABT achieves a much higher degree of translational validity than other behavioural readouts used in preclinical research. The pharmacological data support predictive validity and sensitivity to different pharmacological classes, as well as differentiating mechanistically between conventional delayed onset and RAADs. Comparing the neural mechanisms found to modulate affective biases in the ABT also aligns favourably with human fMRI imaging studies (Hamani et al., 2011: Pizzagalli and Roberts, 2022), suggesting construct validity.

Impairments in reward learning seen in MDD have been modelled in rodents using two different behavioural approaches, the reward learning assay (RLA; Hinchcliffe et al., 2024a, Figure 1) and the probabilistic reward task (PRT, Kangas et al., 2020; Luc and Kangas, 2024). The RLA uses a similar protocol to the ABT, but the animals remain in the same affective state throughout the protocol, and the associative learning involves the pairing of either a high or low value reward with a specific digging substrate (Hinchcliffe et al., 2024a). The animals learn each reward association on a different day, and then a choice test is used where both the reward-paired substrates are presented at the same time, and the animals’ choices are recorded. Normal animals exhibit a reward-induced positive bias, making more choices for the digging substrate previously paired with the high-value reward. When the protocol is run in animals in a putative depression-like state, this reward-induced bias is lost (Figure 1, Hales et al., 2023; Stuart et al., 2019). The RLA has been tested in different animal models of depression, and similar impairments in reward learning are observed. These findings suggest this is a core behavioural deficit observed in animals in a negative affective state and is similar to the reward learning impairments associated with anhedonia in MDD. In contrast to the findings with the SPT, where only chronic stress or chronic corticosterone treatment induces a reliable deficit, in the RLA, impaired reward learning was seen in animals exposed to early life adversity, chronic pro-depressant drug treatment, chronic pro-inflammatory treatment and chronic pain (Robinson, 2018; Phelps et al., 2021). Further studies to explore the neural circuits mediating these effects and sensitivity to antidepressant manipulations have yet to be undertaken, but these behavioural findings suggest this approach has improved translational validity.

The PRT for rodents may quantify similar reward learning impairments as the RLA but is designed to run in the touchscreen apparatus, providing the advantage of being fully automated (Kangas et al., 2020; Luc and Kangas, 2024). The rodent PRT is also very closely aligned to the human PRT, adding further opportunities for validation and strengthening clinical relevance. In the PRT for humans and rodents, the subject is required to make visual discriminations with probabilistic contingencies arranged such that correct responses to one alternative are rewarded more often (rich) than correct responses to the other (lean). As predicted by signal detection theory, healthy participants develop a response bias in favour of the rich alternative without disruption in overall task discriminability. By contrast, patients with MDD exhibit a lower response bias, which correlates with anhedonia (Pizzagalli et al., 2005). Rodents can be trained to perform this task in the touchscreen apparatus and develop a reward-induced bias which is sensitive to pharmacological manipulations (Der-Avakian et al., 2017) and both chronic stress (Kangas et al., 2022a) and early life adversity models of depression (Kangas et al., 2024). Most pharmacological studies in the PRT have focused on the dopamine system with a blunted reward bias observed following treatment with low-dose pramipexole, a dopamine2/3 agonist thought to reduce dopamine levels through presynaptic inhibition (Kangas et al., 2022b). These effects were like those in human participants given the same treatment. The effects of pramipexole were replicated in a separate study, and further characterisation using amphetamine found the opposite effect with an increase in response bias associated with higher levels of dopamine (Lamontagne et al., 2018). Further supporting the evidence that reward bias is sensitive to stress, acute treatment with dexamethasone also blunted reward bias in rats in this task (Lamontagne et al., 2018). The PRT has also been reverse translated for non-human primates (Wooldridge et al., 2021).

## Modelling psychedelic effects and hallucinations in non-human animals

Serotonergic psychedelic drugs induce profound changes in perception, which have been strongly linked to their activity at the 5-HT2A receptors. These effects may limit the wider use of these drugs clinically and will add significant cost implications if licensed for psychiatric disorders. Agonism or partial agonism at 5-HT2A receptors is a common characteristic of serotonergic psychedelics, but other receptors are also modulated by these drugs at clinical doses and may be involved in their antidepressant effects. Furthermore, not all 5-HT2A agonists induce psychedelic effects, with some studies in traditional animal models for MDD suggesting the antidepressant effects do not require the psychedelic effects to achieve efficacy (Cameron et al., 2021). This raises the possibility that drugs with reduced 5-HT2A-mediated psychedelic effects could achieve efficacy with better tolerability and offer a more cost-effective treatment. To test this hypothesis and provide a preclinical pipeline for development, assays that can predict the hallucinatory and altered perceptual effects of novel drugs are needed.

The HTR and WDSs were first described as assays that could detect activation of central serotonin function (Bedard et al., 1977; Corne et al., 1963). Subsequent use of the models expanded, and the HTR is the most widely used animal model for detecting hallucinogenic properties of drugs irrespective of their pharmacological mechanism of action (Alexander et al., 2024; Corne et al., 1963). The problem with the HTR as a universal model to detect psychedelic activity and predict effects in humans is that not all drugs which alter perception in humans induce HTR in rodents, for example, MDMA. There are also limitations with these methods, including the time course of effects, specificity and dose–response relationship (for further discussion, see Alexander et al., 2024). The HTR and WDS induced by psychedelics are a 5-HT2A mediated behaviour in rodents, with 5-HT2A antagonists reliably blocking the behavioural response. These measures are unlikely to capture the full psychedelic experience in humans but may provide a useful assay for in vivo evaluation of 5-HT2A receptor agonism and potentially some insights into biased agonism (Alexander et al., 2024). Similar to the issues relating to symptoms of MDD, the quantification of sensory and perceptual effects of serotonergic psychedelics in human subjects is largely based on self-reported measures such as the Altered States of Consciousness questionnaire. With the quality of the psychedelic effects defined by experiences such as Oceanic Boundlessness and Dread of Ego Dissolution alongside descriptions of sensory perceptual effects, relating these to effects in animals poses significant challenges (Roseman et al., 2018). An alternative approach is the drug-discrimination paradigm where rats are trained to discriminate a psychedelic 5-HT2A agonist such as DOM (2,5-Dimethoxy-4-methylamphetamine). The ability of a test substance to substitute for the trained psychedelic has good predictive validity (Glennon, 1983; Halberstadt, 2020). Drug discrimination methods may therefore offer some benefits, but are still not 100% reliable. Few studies have yet sought to develop new behavioural tasks, but there is potential to develop translational tasks of perception based on cue-discrimination tasks. In a recent preliminary study, a novel visual perception task was able to quantify psilocybin-induced perceptual changes in human subjects and rats (Vejmola et al., 2025). The task was based on the animal’s ability to detect and discriminate between different visual cues, with the drug-induced changes in visual perception resulting in impaired accuracy in the task. A control for general impairments was included using a pair of cues, which were easily discriminated from each other even under drug treatment. Although still only in the early stages of development, this task and associated testing in human and non-human subjects illustrate the potential to develop relevant translational approaches to quantify the psychedelic effects of novel drugs.

## Summary

Although many studies still utilise traditional behavioural methods to quantify depression-related behaviours, antidepressant mechanisms and novel drug targets, the repeated failure of findings using these methods to translate to clinical benefits cannot be ignored. The tasks discussed in this article offer new opportunities for preclinical studies associated with the serotonergic psychedelics and MDD, with a focus on ‘behavioural biomarkers’ which can be quantified in human and non-human animals using objective methods. These approaches have shown improved translational validity, particularly in relation to studies investigating the antidepressant effects of psychedelics, where traditional behavioural methods are limited. Integrating these tasks with circuit and molecular studies is starting to reveal new insights into the possible mechanisms that link their biochemical effects with relevant behavioural domains and core symptoms associated with MDD, such as low mood and anhedonia.

## Notes

1. The terms ‘control animals/control condition’ or ‘untreated animals’ are used in this article as opposed to ‘normal controls’ to reflect the potential impact of unnatural laboratory housing and management systems on the affective state of the animals.
